# Idiopathic pulmonary arteriovenous malformation: a rarity in clinical practice

**DOI:** 10.1590/1677-5449.202400052

**Published:** 2025-01-13

**Authors:** Tarcila Gurgel Aquino, Diogenes de Melo Jacó, Ingryd Gabriella Nascimento Santos, Eliauria Rosa Martins

**Affiliations:** 1 Universidade Federal da Paraíba – UFPB, Hospital Universitário Lauro Wanderley – HULW, João Pessoa, PB, Brasil.

**Keywords:** arteriovenous malformations, pulmonary arteriovenous malformations, arteriovenous malformations/epidemiology, arteriovenous malformations/clinical manifestations, arteriovenous malformations/diagnosis, arteriovenous malformations/treatment

## Abstract

Pulmonary arteriovenous malformations (PAVM) are characterized by abnormal pulmonary vessels forming arteriovenous shunts that compromise oxygenation of the blood, causing hypoxemia, and predispose to infections and cerebral ischemia. The patient in this case was a 38-year-old male who presented with tachypnea and dyspnea, cyanosis of extremities, and significant digital clubbing. The patient had structural epilepsy secondary to neurosurgery for a cerebral abscess during childhood. Arterial blood gas analysis showed significant hypoxemia (PaO_2_ = 46.2; SaO_2_ = 77%; PaO_2_/FiO_2_ = 70) and a chest computed tomography showed PAVM in the apical segments of the right upper and lower lobes, with ectatic and tortuous vascular structures following an intraparenchymal path, communicating with the pulmonary artery and veins. After confirmation of the PAVM, it was concluded that elevated pulmonary resistance was contributing to refractive hypoxemia and hypercapnia. Gradual reduction of the ventilation parameters, primarily controlled pressure and positive end-expiratory pressure, and consequent reduction of the arteriovenous shunt, resulted in progressive improvement of oxygenation and respiratory mechanics. The vascular surgery team’s assessment was that treatment with embolization was warranted.

## INTRODUCTION

Pulmonary arteriovenous malformations (PAVM) are characterized by abnormal pulmonary vessels and occur when an artery connects directly to a vein, forming an arteriovenous shunt,^1^ with blood passing directly from the right side of the heart to the left side, without communication via capillaries. This compromises oxygenation of the blood and can cause hypoxemia and a series of other clinical manifestations, such as predisposition to infections (cerebral, hepatic, and/or splenic abscesses) and ischemic stroke, because the absence of capillary filtration enables bacteria and thrombi to pass directly from the venous system to the arterial system.

This case report will describe the clinical and radiological manifestations and course of a patient with an idiopathic PAVM (iPAVM) who was admitted to the Hospital Universitário Lauro Wanderley in the city of João Pessoa, Paraíba, Brazil, highlighting the signs and symptoms suggestive of chronic hypoxemia that supported the clinical diagnosis. This report will also discuss the treatment options, emphasizing immediate initiation of therapeutic measures as an important factor in prognosis, in addition to calling the attention of the medical community to the potential severity of the etiology in question.

This study was approved by the Research Ethics Committee at the Hospital Universitário Lauro Wanderley (HULW) at the Universidade Federal da Paraíba (UFPB), after acceptance of the free and informed consent form, with Ethics Appraisal Submission Certificate number 63397522.4.0000.5188 and consolidated opinion number 5.808.822.

## CASE REPORT

**Figure 1:**
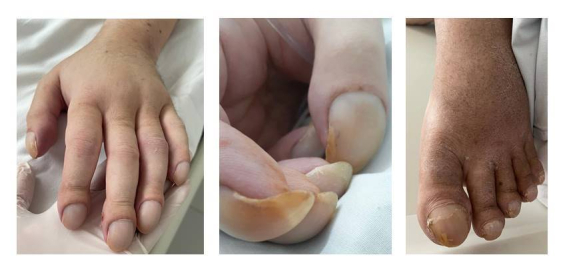
Digital clubbing.

The patient was a 38-year-old male who had a history of difficult to control convulsive crises since childhood and arrived at the intensive care unit (ICU) with tachypnea and dyspnea and cyanosis of the extremities, in addition to significant nail clubbing (Figure 1). He was breathing spontaneously through a mask with a 15 liters per minute reservoir and pulse oximetry was showing peripheral oxygen saturation (SpO2) of 80%. There were no reports of a need for prior admissions or episodes of acute respiratory failure and the patient denied orthopnea, trepopnea, platypnea, and paroxysmal nocturnal dyspnea. Arterial blood gas analysis was conducted on admission, showing significant hypoxemia (PaO_2_ = 46.2; SaO_2_ = 77%; PaO_2_/FiO_2_ = 70 mmHg).

**Figure 2:**
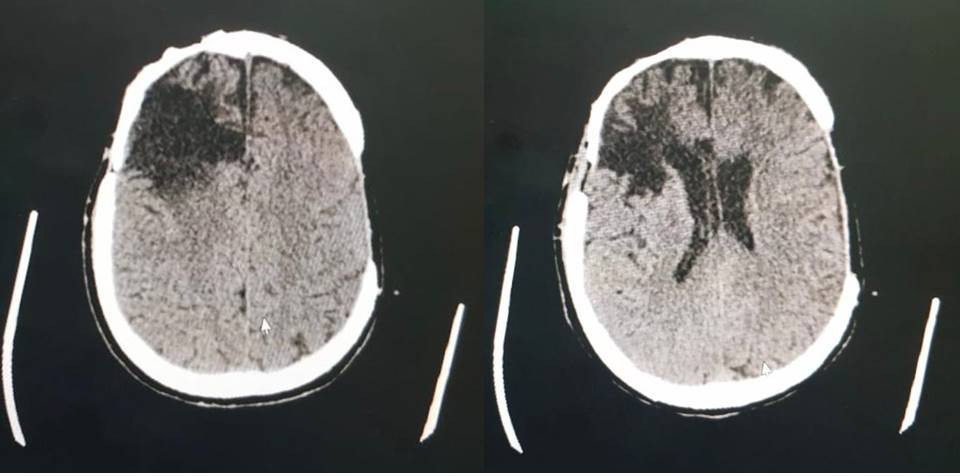
Computed tomography of the head without contrast, showing hypoattenuation of the right cortico-subcortical frontal and left temporal regions and ectasia of adjacent cortical sulci and lateral ventricles, suggestive of encephalomalacia/gliosis.

Work-up was initiated with computed tomography (CT) of the head without contrast (Figure 2), showing hypoattenuation of the right cortico-subcortical frontal and left temporal regions and ectasia of adjacent cortical sulci and lateral ventricles, suggestive of encephalomalacia/gliosis, compatible with the patient’s family’s report of neurosurgery to treat a cerebral abscess at 9 years of age, explaining the patient’s neurological status at admission, and characteristic of structural epilepsy secondary to surgical manipulation. A chest CT without contrast showed oval nodular formations of a serpentine appearance, with pervasive large-caliber vascular structures conflating at the pulmonary hilum, most noticeably in the apical segment of the upper right lobe (URL), measuring 7.0 x 6.1cm, and the apical segment of the right lower lobe (RLL), measuring 9.7 x 4.7cm. These formations were associated with fibroreticular, micronodular opacities and pervasive ground-glass attenuation, which could indicate PAVM, intralobar pulmonary sequestration, or an association with inflammatory/infectious lung disease, which are indistinguishable using this method. The patient’s condition deteriorated, progressing to SpO_2_ = 57%, central cyanosis, and impaired level of consciousness, and oral endotracheal intubation was performed. Post-procedural blood gas analysis showed pH = 7.45; PaO_2_ = 49.3; PaCO_2_ = 48.6; SaO_2_ = 79%; HCO_3_ = 34.1; and PaO_2_/FiO_2_ = 141.

**Figure 3:**
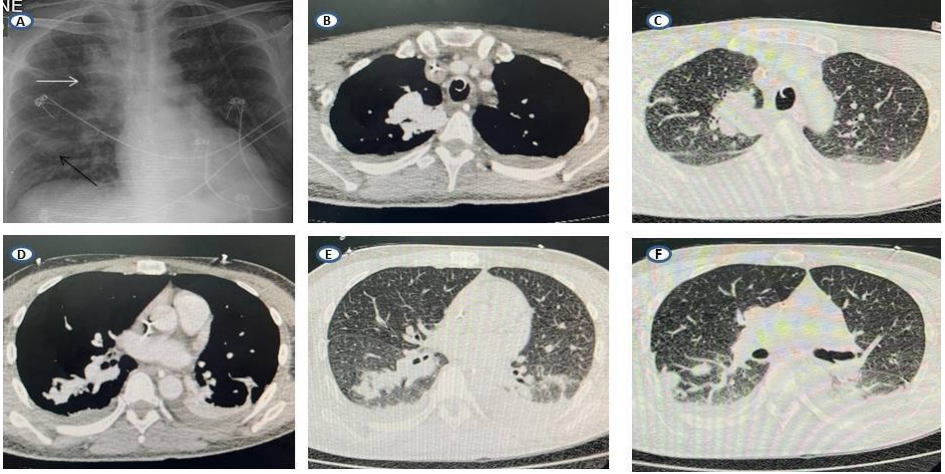
Pulmonary radiological examination. In **A)** chest X-ray showing arteriovenous malformation (AVM) in the apical segment of the upper right lobe (URL) – white arrow – and the apical segment of the right lower lobe (RLL) – black arrow; in **B** and **C)** computed tomography (CT) of the chest with contrast showing AVM in the URL; and in **D, E,** and **F)** chest CT with contrast showing AVM in the RLL and the apical segment of the left lower lobe.

**Figure 4:**
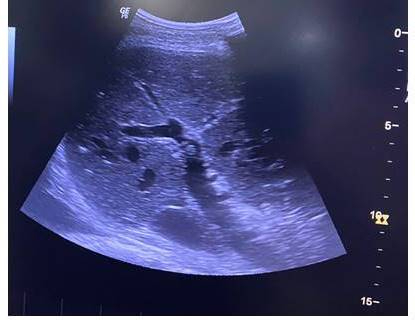
Hepatic arteriovenous malformations seen on upper abdominal ultrasonography.

**Figure 5:**
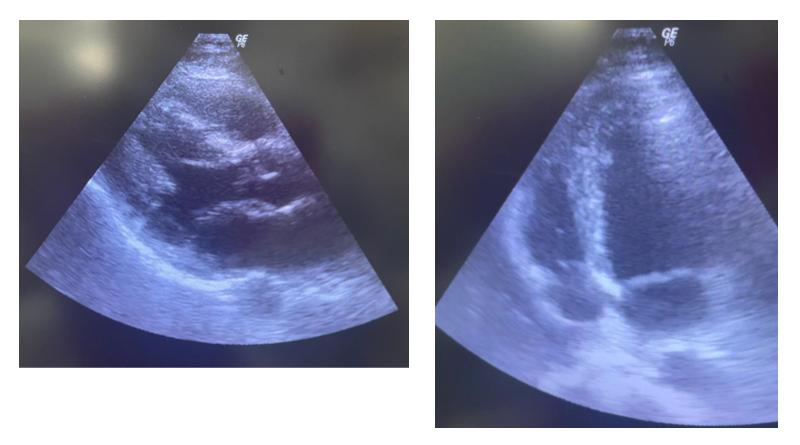
Transthoracic echocardiography with no direct signs of pulmonary arterial hypertension.

After clinical stabilization, the patient was sent for chest CT with contrast (Figure 3), showing a right-side pulmonary vascular malformation, with ectatic and tortuous structures vascular following an intraparenchymal path in the upper segments of the RLL and URL and communicating with the pulmonary artery and veins on the right, suggesting an arteriovenous malformation (AVM). The patient also underwent abdominal CT with contrast, showing the liver with normal morphology and outlines, but diffuse enlargement of dimensions and heterogeneous peripheral areas, possibly caused by arteriovenous shunts and suggesting the possibility of hereditary hemorrhagic telangiectasia (HHT). These hepatic changes were also observable on upper abdominal ultrasonography (Figure 4). Transthoracic echocardiograms (Figure 5) were performed by three specialists at different times and showed no indirect signs of pulmonary arterial hypertension (PAH), such as enlarged right cardiac chambers, paradoxical interventricular septal movement, tricuspid valve reflux, abnormal tricuspid annular plane systolic excursion, or elevated systolic pulmonary artery pressure. However, there were technical difficulties with conducting the examination at the bedside because of the patient’s position, preventing measurement of deformation of the myocardial tissue and fractional area change of the right ventricle.

The patient developed refractory septic shock secondary to mechanical ventilation-induced pneumonia and it was necessary to institute broad spectrum antibiotic therapy guided by tracheal secretion culture, in addition to infusion of vasoactive drugs and corticoid therapy.

Once the diagnostic suspicion of AVM had been raised, it was hypothesized that the elevated pulmonary resistance could be contributing to the refractory hypoxemia and hypercapnia. In view of this, the ventilator parameters were reduced gradually, in particular the controlled pressure and alveolar positive end-expiratory pressure, which, in addition to the consequent reduction in the arteriovenous shunt, led to progressive improvement in oxygenation and respiratory mechanics, culminating in a successful spontaneous respiration test and extubation. The patient was assessed by the vascular surgery team, which recommended embolization to treat the PAVM. The patient was transferred to a center of excellence in endovascular procedures for treatment. The embolization images were only displayed and analyzed during the procedure and were not recorded and are not therefore available for analysis.

## DISCUSSION

One of the most frequent etiologies of PAVM is HHT, also known as de Rendu-Osler-Weber syndrome.^2^ Diagnosis is primarily based on the International Clinical Diagnosis Criteria (Curaçao Criteria de), which consider the following clinical and radiological variables: 1) multiple telangiectases in typical sites, primarily face, lips, hands, and oral cavity; 2) recurrent nosebleeds (epistaxis); 3) AVM with visceral involvement (cerebral, pulmonary, hepatic, gastrointestinal, or spinal cord); and 4) family history – first-degree relative.^3^ The diagnosis is considered definitive for HHT if three or more of these criteria are present. Those who do not meet at least two of these conditions are classified as having iPAVM. According to this classification, our patient scores just one point (visceral arteriovenous malformations), so the diagnosis is possible, but improbable.^4^ Hereditary hemorrhagic telangiectasia is responsible for 80 to 95% of cases of PAVM and PAVM is classified as idiopathic in the absence of clear definition of this syndrome. Since it is an under-diagnosed disease, many patients are unaware of the etiology of their disease for decades, as illustrated by the case reported here. No prior investigation had been conducted, despite the significant digital clubbing and signs of chronic hypoxemia.

Female patients and fistulas located in the lower pulmonary lobes predominate.^1^ Multiple PAVMs are more common in HHT, making these patients more symptomatic.^1^ However, the majority of iPAVM cases have single fistulas with larger caliber and hypoxemia without dyspnea, which diverges from the case reported here.^1^ One interesting finding is that patients with low SpO_2_ have few symptoms and only report dyspnea during significant effort, which is related to the period of adaptation to hypoxemia.

Presence of PAH is rare^4^ and when present it may be because of increased flow in pulmonary vessels secondary to hepatic AVM or because of anemia. In the case in question, the patient had AVM in the liver and nevertheless did not develop PAH. However, he did develop polycythemia, as a form of adaptation to and compensation for chronic hypoxemia.

Rupture of PAVM is a rare complication, except in pregnancy.^5^ Treatment for PAVMs is recommended even in oligosymptomatic or asymptomatic patients, with the objective of avoiding the risks of serious and potentially fatal complications.^5^

In the past, PAVMs were treated surgically, with high rates of complications.^6^ Until the end of the 1970s, the procedures of choice for pulmonary AVPs were pulmonary lobectomy, wedge resection of the segment involved, or direct surgical ligation of the arteriovenous fistula.^3^ Nowadays, pulmonary embolization via percutaneous catheterization has become the standard treatment,^3^ which is a much less invasive procedure and reduces the risk of postoperative complications secondary to partial or total lobectomy and shortens the length of hospital stay.^5^ Fistulas with a feeder artery diameter exceeding 3 mm should be embolized even in asymptomatic patients, in order to avoid infectious and vascular complications.^6^

Percutaneous pulmonary embolization uses the anchor coil technique, which consists of placing a coil into a small collateral branch of the main feeder artery, immediately upstream of the arteriovenous malformation, enabling ideal occlusion of the cross-section and avoiding accidental movement of the device and distal migration to the left circulation.^5^

When all of the arteries are obliterated, the sac regresses within 6 months of intervention. However, if a feeder artery is not embolized, the fistula sac may not regress, indicating a possibility of recanalization.^5^ Presence of blood flow remains after embolization in around 25% of cases, caused by recanalization, when flow is via the embolized fistula, or by reperfusion, when an accessory artery is ruptured.^5^ In both scenarios the treatment of choice is repeat embolization.^5^

Embolization may be associated with benign complications, in particular pain and pleural effusion, which improve with symptomatic treatment. Other important complications that are seen more rarely include symptomatic pulmonary infarction and systemic migration of the device across the AVM, which can be avoided by careful placement and criteria-guided choice of coil size. Other complications such as gas emboli, transitory angina, cardiac arrhythmia, deep venous thrombosis, and pneumothorax have been reported at even lower frequencies.^3^

Nowadays, surgical intervention is reserved for cases in which percutaneous treatment is unsuccessful, when feeder arteries have very large caliber, or when the vascular anatomy is unfavorable for treatment by embolization.^6^ Lung transplantation is a treatment option for exceptional cases only,^6^ since, despite the hypoxemia and risks of infection or ischemia related to the disease, survival of these patients is often better than survival after lung transplantation.

In view of the above, it is clear that clinical diagnosis is a significant challenge to medical practice, since the need for high complexity imaging exams is essential in the process of etiological investigation, which can be an obstacle because of access difficulties. Moreover, there is the possibility that patients will adapt to chronic hypoxemia, and are very often oligosymptomatic or asymptomatic, which could delay diagnosis because of a low degree of clinical suspicion. It is therefore of fundamental importance to make the medical community aware of this situation so that this condition is included in differential diagnosis of chronic hypoxemia, enabling early diagnosis and immediate treatment, thereby reducing chronic damage and improving patient quality of life.
