# Supplementary material for: Idiopathic pulmonary arteriovenous malformation: a rarity in clinical practice
Source: J Vasc Bras. 2025 Jan 13;23:e20240005. doi: 10.1590/1677-5449.202400052 (PMC11758929; doi:10.1590/1677-5449.202400052)
Supplement: Translation

# Translation: Malformação arteriovenosa pulmonar idiopática: raridade na prática clínica

**DOI:** 10.1590/1677-5449.202400051

**Authors:** Tarcila Gurgel Aquino, Diogenes de Melo Jacó, Ingryd Gabriella Nascimento Santos, Eliauria Rosa Martins

**Affiliations:** 1 Universidade Federal da Paraíba – UFPB, Hospital Universitário Lauro Wanderley – HULW, João Pessoa, PB, Brasil.

**Keywords:** malformações arteriovenosas, malformações arteriovenosas pulmonares, malformações arteriovenosas/epidemiologia, malformações arteriovenosas/manifestações clínicas, malformações arteriovenosas/diagnóstico, malformações arteriovenosas/tratamento

## Abstract

As malformações arteriovenosas pulmonares (MAVP) são caracterizadas por vasos pulmonares anormais, formando *shunts* arteriovenosos que comprometem a oxigenação sanguínea e ocasionam hipoxemia, além de predisposição a infecções e isquemia cerebral. O paciente, de 38 anos, do sexo masculino, apresentou-se com quadro de taquidispneia, cianose de extremidades e hipocratismo digital importante. Foi referido epilepsia estrutural pós-intervenção neurocirúrgica devido a um abscesso cerebral durante a infância. A gasometria arterial constatou hipoxemia importante (PaO_2_ = 46,2; SaO_2_ = 77%; PaO_2_/FiO_2_ = 70) e, na tomografia computadorizada de tórax, foi observada MAVP no segmento apical dos lobos superior e inferior direitos, com estruturas vasculares ectasiadas e tortuosas apresentando trajeto intraparenquimatoso e mantendo relação com artéria e veias pulmonares. Após comprovação de MAVP, percebeu-se que a resistência pulmonar elevada contribuía para a hipoxemia e hipercapnia refratárias. Assim, a redução gradual dos parâmetros ventilatórios, sobretudo da pressão controlada e da pressão expiratória final positiva, e, consequentemente, do *shunt* arteriovenoso, resultou na melhora progressiva da oxigenação e mecânica ventilatória. A avaliação da equipe da cirurgia vascular indicou tratamento via embolização.

## INTRODUÇÃO

As malformações arteriovenosas pulmonares (MAVP) são caracterizadas por vasos pulmonares anormais e ocorrem quando uma artéria se conecta diretamente a uma veia, formando um *shunt* arteriovenoso^1^ , onde há passagem direta do sangue do lado direito do coração para o esquerdo sem a comunicação pelo capilar. Isso compromete a oxigenação sanguínea, podendo ocasionar hipoxemia e uma série de diferentes manifestações clínicas, como predisposição a infecções (abscesso cerebral, hepático e/ou esplênico) e acidente vascular cerebral isquêmico, pois a perda do filtro capilar permite a passagem de bactérias e trombos diretamente do sistema venoso para o arterial.

O relato de caso descreverá as manifestações clínico-radiológicas e evolutivas de um paciente com MAVP idiopática (MAVPi) que esteve internado no Hospital Universitário Lauro Wanderley, na cidade de João Pessoa, Paraíba, salientando as alterações semiológicas sugestivas de hipoxemia crônica que favoreceram o diagnóstico clínico. Ademais, este relato discutirá as opções de tratamento, enfatizando o início imediato das medidas terapêuticas como fator importante no prognóstico, além de chamar atenção da comunidade médica para a potencial gravidade da etiologia em questão.

Este estudo foi aprovado pelo Comitê de Ética em Pesquisa do Hospital Universitário Lauro Wanderley (HULW) da Universidade Federal da Paraíba (UFPB), mediante aceitação do Termo de Consentimento Livre e Esclarecido, tendo o Certificado de Apresentação para Apreciação Ética de número 63397522.4.0000.5188, e o seguinte número do Parecer Consubstanciado: 5.808.822.

## RELATO DE CASO

O paciente, de 38 anos, do sexo masculino, que possuía histórico de crises convulsivas de difícil controle desde a infância, chegou à unidade de terapia intensiva (UTI) contactuante, com quadro de taquidispneia e cianose de extremidades, além de hipocratismo digital importante (Figura 1). Respirava espontaneamente sob máscara com reservatório de 15 litros por minuto e com oximetria de pulso marcando 80% de saturação periférica de oxigênio (SpO2). Não tendo relato de necessidade de internações prévias ou episódios de insuficiência respiratória aguda, o paciente negou ortopneia, trepopneia, platipneia e dispneia paroxística noturna. Foi realizada gasometria arterial da admissão, que constatou hipoxemia importante (PaO_2_ = 46,2; SaO_2_ = 77%; PaO_2_/FiO_2_ = 70 mmHg).

**Figura 1:** Hipocratismo digital.

A investigação do caso foi iniciada com a realização da tomografia computadorizada (TC) de crânio sem contraste (Figura 2), que evidenciou: hipoatenuação corticossubcortical frontal direita e temporal esquerda associada à ectasia de sulcos corticais e ventrículos laterais adjacentes, sugestivas de encefalomalácia/gliose, sendo tal achado compatível com o relato de abordagem neurocirúrgica de abscesso cerebral aos 9 anos de idade relatada pela família, justificando o quadro neurológico admissional do paciente, característico de epilepsia de causa estrutural após manipulação cirúrgica. Na TC de tórax sem contraste, foi visto: formações nodulares ovaladas de aspecto serpiginoso com estruturas vasculares calibrosas de permeio, confluindo ao hilo pulmonar, destacando-se no segmento apical do lobo pulmonar superior direito (LSD), medindo 7,0 x 6,1cm, e no segmento apical do lobo pulmonar inferior direito (LID), medindo 9,7 x 4,7cm. Essas formações estavam associadas a opacidades fibrorreticulares, micronodulares e com atenuação em vidro fosco de permeio, podendo significar MAVP, sequestro pulmonar intralobar ou associação com pneumopatia inflamatória/infecciosa, indistinguíveis ao método. O paciente apresentou piora do quadro inicial, evoluindo com SpO2 = 57%, cianose central e rebaixamento do nível de consciência, sendo prosseguida a intubação orotraqueal. A gasometria pós-procedimento revelou pH = 7,45; PaO_2_ = 49,3; PaCO_2_ = 48,6; SaO_2_ = 79%; HCO_3_ = 34,1; PaO_2_/FiO_2_ = 141.

**Figura 2:** Tomografia computadorizada de crânio sem contraste com hipoatenuação corticossubcortical frontal direita e temporal esquerda, associada à ectasia de sulcos corticais e ventrículos laterais adjacentes, sugestivas de encefalomalácia/gliose.

Após estabilização clínica, o paciente foi submetido à TC de tórax com contraste (Figura 3), onde foi visualizado malformação vascular pulmonar à direita, com estruturas vasculares ectasiadas e tortuosas, com trajeto intraparenquimatoso no segmento superior do LID e LSD, mantendo relação com a artéria e veias pulmonares à direita, sugerindo malformação arteriovenosa (MAV). Ademais, o paciente foi submetido à TC de abdome com contraste, que revelou fígado com morfologia e contornos preservados, exibindo aumento difuso de suas dimensões e áreas heterogêneas em sua periferia, possivelmente decorrentes de *shunts* arteriovenosos, sugerindo a possibilidade de telangiectasia hemorrágica hereditária (THH). As alterações hepáticas também foram percebidas na ultrassonografia abdominal superior (Figura 4). No ecocardiograma transtorácico (Figura 5), realizado por três especialistas em momentos distintos, não foram identificados sinais indiretos de hipertensão arterial pulmonar (HAP), como: aumento de câmaras cardíacas direitas, movimento paradoxal do septo interventricular, refluxo valvar tricúspide, alteração na medida de excursão sistólica do plano do anel tricúspide e elevação da pressão sistólica da artéria pulmonar. No entanto, houve dificuldade técnica para a realização do exame à beira do leito devido ao posicionamento do enfermo, impossibilitando a medição da deformação do tecido miocárdico e da variação fracional da área do ventrículo direito. O paciente desenvolveu choque séptico refratário em decorrência à pneumonia associada à ventilação mecânica, sendo necessário uso de antibioticoterapia de largo espectro guiado por cultura de secreção traqueal, além de infusão de droga vasoativa e corticoterapia.

**Figura 3:** Exames radiológicos pulmonares. Em **A)** radiografia de tórax mostrando malformação arteriovenosa (MAV) em segmento apical do lobo pulmonar superior direito (LSD) – seta branca – e no segmento apical do lobo pulmonar inferior direito (LID) – seta preta; em **B** e **C)** tomografia computadorizada (TC) de tórax com contraste evidenciando MAV em LSD; e em **D, E** e **F)** TC de tórax com contraste evidenciando MAV em LID e no segmento apical do lobo pulmonar inferior esquerdo.

**Figura 4:** Malformações arteriovenosas hepáticas visualizadas em ultrassonografia de abdome superior.

**Figura 5:** Ecocardiografia transtorácica sem sinais indiretos de hipertensão arterial pulmonar.

Após a suspeita diagnóstica de MAV, aventou-se a possibilidade de a resistência pulmonar elevada contribuir para a hipoxemia e hipercapnia refratárias. Assim, a redução gradual dos parâmetros ventilatórios, especialmente a pressão controlada e a pressão expiratória final positiva alveolar, além da consequente redução do *shunt* arteriovenoso, resultou na melhora progressiva da oxigenação e da mecânica ventilatória, culminando no sucesso do teste respiratório espontâneo e posterior extubação. Após avaliação pela equipe da cirurgia vascular, foi indicada a embolização como forma de tratamento para MAVP. Então, o paciente foi transferido para o serviço de referência de procedimentos endovasculares do estado para dar seguimento à terapêutica do caso. As imagens da embolização foram realizadas e analisadas apenas durante o procedimento, não ficando registradas para apreciações futuras.

## DISCUSSÃO

Uma das principais etiologias para MAVP é a THH, também conhecida como síndrome de Rendu-Osler-Weber^2^ . Para o seu diagnóstico, é utilizado principalmente o Critério Internacional Clínico de Diagnóstico (Critério de Curaçao), que considera as seguintes variáveis clínicas e radiológicas: 1) telangiectasias múltiplas em locais típicos, principalmente em face, lábios, mãos e cavidade oral; 2) epistaxes recorrentes; 3) MAV com comprometimento visceral (cerebral, pulmonar, hepática, gastrointestinal ou medular); e 4) história familiar – parente de primeiro grau^3^ . O diagnóstico é considerado como definitivo de THH com o encontro de três ou mais desses critérios. Aqueles que não preencheram pelo menos duas dessas condições são classificados como portadores de MAVPi. De acordo com essa classificação, nosso paciente pontua apenas um ponto (malformações arteriovenosas viscerais), enquadrando-se entre aqueles com diagnóstico possível, mas improvável^4^ . A THH é responsável por 80 a 95% dos casos de MAVP e, quando não se tem a caracterização clara da síndrome, as MAVP são classificadas como idiopáticas. Por ser uma doença subdiagnosticada, vários pacientes permanecem por décadas sem saber a etiologia da doença, como foi exemplificado neste relato. Apesar do importante baqueteamento digital e dos sinais de hipoxemia crônica, não havia sido feita uma investigação anterior.

Observa-se uma predominância no sexo feminino e de fístulas localizadas nos lobos pulmonares inferiores^1^ . Múltiplas MAVPs são mais comuns nas THH, tornando os indivíduos mais sintomáticos^1^ . Por outro lado, a maior parte das MAVPis apresenta fístulas únicas de maior calibre e hipoxemia sem dispneia, o que diverge do caso exposto neste relato^1^ . Um fato interessante é que pacientes com SpO2 reduzida são pouco sintomáticos e referem apenas dispneia durante grandes esforços, relacionando-se ao período de adaptação à hipoxemia.

A presença de HAP é rara^4^ e, quando presente, pode ser secundária ao aumento do fluxo dos vasos pulmonares em decorrência de MAV hepática ou por anemia. No caso em questão, o paciente possui MAV no fígado e, apesar disso, não desenvolveu HAP. Ademais, apresentava poliglobulia, como forma de adaptação e compensação à cronicidade hipoxêmica.

A ruptura de MAVP é uma complicação rara, exceto na gravidez^5^ . O tratamento das MAVPs foi proposto mesmo em pacientes oligo ou assintomáticos, com o intuito de evitar os riscos de complicações graves e potencialmente fatais^5^ .

No passado, as MAVPs eram tratadas cirurgicamente, com uma taxa expressiva de complicações^6^ . Até o final da década de 1970, os procedimentos de escolha para as MAVs pulmonares eram lobectomia pulmonar, ressecção em cunha do segmento acometido ou ligadura cirúrgica direta da fístula arteriovenosa^3^ . Hoje em dia, a embolização pulmonar por cateter por via percutânea tornou-se a terapia padrão^3^ , sendo um procedimento bem menos invasivo e diminuindo o risco de complicações pós-operatórias secundárias à lobectomia parcial ou total, com redução do tempo de internação hospitalar^5^ . As fístulas devem ser embolizadas mesmo em pacientes assintomáticos, desde que tenham um diâmetro da artéria nutridora maior que 3 mm, a fim de se evitar complicações infecciosas e vasculares^6^ .

A embolização pulmonar percutânea utiliza a técnica de ancoragem de molas, que consiste em travar a mola dentro de um pequeno ramo colateral da principal artéria nutridora, imediatamente a montante da malformação arteriovenosa, permitindo a oclusão ideal da seção transversal e evitando a maior mobilização acidental do dispositivo e sua migração distal para a circulação esquerda^5^ .

Quando todas as artérias são obliteradas, o saco regride dentro de 6 meses após a intervenção. Entretanto, se alguma artéria nutridora não for embolizada, o saco fistuloso pode não regredir, sinalizando a possibilidade de recanalização^5^ . A presença do fluxo sanguíneo após a embolização persiste em cerca de 25% dos casos, ocorrendo por recanalização, quando o fluxo ocorre pela fístula embolizada, ou reperfusão, devido à ruptura de artéria acessória^5^ . Nos dois cenários, o tratamento de escolha é uma nova embolização^5^ .

A embolização pode evoluir com complicações benignas, destacando-se dor e derrame pleural, que melhoram ao ser instituído o tratamento sintomático. Outras complicações importantes e mais raramente encontradas são: infarto pulmonar sintomático e migração sistêmica do dispositivo através da MAV, as quais podem ser evitadas pelo posicionamento cuidadoso e pela escolha criteriosa do tamanho das molas. Ainda, em menor quantidade, foram relatadas a embolia gasosa, a angina transitória, a arritmia cardíaca, a trombose venosa profunda e o pneumotórax^3^ .

Atualmente, a intervenção cirúrgica fica restrita a casos de insucesso com o tratamento percutâneo, quando as artérias nutridoras apresentam calibres muito grandes ou quando a anatomia vascular não favorece o tratamento por embolização^6^ . O transplante de pulmão constitui-se em um recurso terapêutico de exceção^6^ , visto que, apesar da hipoxemia e dos riscos infecciosos ou isquêmicos relacionados à doença, a sobrevida desses pacientes é frequentemente maior do que nos indivíduos transplantados.

Diante do exposto, torna-se evidente que o diagnóstico clínico representa um grande desafio na prática médica, uma vez que a necessidade de exames de imagem de alta complexidade faz-se essencial no processo de investigação etiológica, podendo ser um obstáculo devido à dificuldade de acesso. Além disso, existe a possibilidade do paciente adaptar-se à hipoxemia crônica, sendo muitas vezes oligo ou assintomático, o que pode retardar o diagnóstico em razão da baixa suspeição clínica. Portanto, é fundamental alertar a comunidade médica acerca dessa situação, para que se possa estabelecer tal condição como diagnóstico diferencial de hipoxemia crônica, permitindo diagnóstico precoce e tratamento imediato, reduzindo danos crônicos e melhorando a qualidade de vida dos pacientes.
